# Non-Destructive Classification of Diversely Stained *Capsicum annuum* Seed Specimens of Different Cultivars Using Near-Infrared Imaging Based Optical Intensity Detection

**DOI:** 10.3390/s18082500

**Published:** 2018-08-01

**Authors:** Jyothsna Konkada Manattayil, Naresh Kumar Ravichandran, Ruchire Eranga Wijesinghe, Muhammad Faizan Shirazi, Seung-Yeol Lee, Pilun Kim, Hee-Young Jung, Mansik Jeon, Jeehyun Kim

**Affiliations:** 1 Department of Electronics and Communication, Faculty of Engineering, Christ (Deemed to be University), Bangalore 560029, India; jyothsna.km@mtech.christuniversity.in; 2 Kyungpook National University, College of IT Engineering, School of Electronics Engineering, 80, Daehak-ro, Buk-gu, Daegu 41566, Korea; nareshr.9169@gmail.com (N.K.R.); jeehk@knu.ac.kr (J.K.); 3 Department of Biomedical Engineering, College of Engineering, Kyungil University, 50, Gamasil-gil, Hayang-eup, Gyeongsan-si, Gyeongsangbuk-do 38428, Korea; eranga@kiu.kr; 4 Center of Medical Physics and Biomedical Engineering, Medical University of Vienna, Waehringer Guertel 18-20, 1090 Vienna, Austria; muhammad.shirazi@meduniwien.ac.at; 5 School of Applied Biosciences, Kyungpook National University, 80, Daehak-ro, Buk-gu, Daegu 41566, Korea; leesy1985@gmail.com; 6 Institute of Biomedical Engineering, Kyungpook National University, 680, Gukchaebosang-ro, Jung-gu, Daegu 41944, Korea; pukim@knu.ac.kr

**Keywords:** swept-source OCT, *Capsicumannuum*, dye staining, depth scan analysis

## Abstract

The non-destructive classification of plant materials using optical inspection techniques has been gaining much recent attention in the field of agriculture research. Among them, a near-infrared (NIR) imaging method called optical coherence tomography (OCT) has become a well-known agricultural inspection tool since the last decade. Here we investigated the non-destructive identification capability of OCT to classify diversely stained (with various staining agents) *Capsicum annuum* seed specimens of different cultivars. A swept source (SS-OCT) system with a spectral band of 1310 nm was used to image unstained control *C. annuum* seeds along with diversely stained *Capsicum* seeds, belonging to different cultivar varieties, such as *C. annuum* cv. PR Ppareum, *C. annuum* cv. PR Yeol, and *C. annuum* cv. Asia Jeombo. The obtained cross-sectional images were further analyzed for the changes in the intensity of back-scattered light (resulting due to dye pigment material and internal morphological variations) using a depth scan profiling technique to identify the difference among each seed category. The graphically acquired depth scan profiling results revealed that the control specimens exhibit less back-scattered light intensity in depth scan profiles when compared to the stained seed specimens. Furthermore, a significant back-scattered light intensity difference among each different cultivar group can be identified as well. Thus, the potential capability of OCT based depth scan profiling technique for non-destructive classification of diversely stained *C. annum* seed specimens of different cultivars can be sufficiently confirmed through the proposed scheme. Hence, when compared to conventional seed sorting techniques, OCT can offer multipurpose advantages by performing sorting of seeds in respective to the dye staining and provides internal structural images non-destructively.

## 1. Introduction

The *Capsicum annum* Pepper (*Capsicum* spp.), belonging to the family of Solanaceae, is widely cultivated and used as a fresh vegetable and fruit. There are mainly five domesticated varieties of *Capsicum* species, which are *Capsicum annuum*, *Capsicum chinense*, *Capsicum baccatum*, *Capsicum frutescens*, and *Capsicum pubescens* [1]. Among them, *Capsicum annuum* (the common bell pepper) is the most commonly cultivated species. These different varieties are extensively cultivated by virtue of its numerous functional properties as antimutagenic, antitumoral, antioxidant, and analgesic [2,3]. Seeds are considered as the fundamental unit of production for the world’s food crop, and therefore, making them resistant to diseases caused by fungi, bacteria, viruses, nematodes, and abiotic stresses, is of primary importance as it affects gainful development and seed generation [4].

Several seed treatments such as seed dressing, seed coating and pelleting are performed to protect the seeds from these kinds of external influences [5]. Common and widely used seed treatment procedures involve the process of treating or coating the seeds with biological, physical, or other chemical agents. Among them, chemical treatment methods are most commonly practiced due to their ability to control plant diseases and pests in less time with automatic treatment machinery [6]. These treatment procedures are immediately followed by seed dyeing, where the seeds are colored with artificial colors to differentiate them from the unstained ones. Colorants may be simple dyes or pigments or more complex hybrid multicomponent colorants that provide specific impacts or offer a potential for keen usefulness [7]. Many vegetable and flower seeds are sold with color coating on them. These colorants are applied on the seeds to differentiate them between genetic trends, brands, applications, and technology. Several agriculturalists also use this dye treatment method to differentiate seeds from different brands or genetic traits [8,9]. Furthermore, these types of dyeing prevent their unintended use as food, animal feed or for the extraction of oil from seeds [10,11,12,13,14].

In large scale agricultural industries, the color sorting process of seeds involves the differentiation of seed categories based on their outer color. The most conventional method practiced for differentiating the dye stained samples was through the visual inspection method. This manual inspection method is highly time-consuming, labor intensive, and subject to human error. These shortcomings of the manual inspection method were nullified by the introduction of electronic sorting machines and color sorting using machine vision techniques, which were used for efficient sorting with reduced labor cost [15]. These systems work by analyzing the shape, size, and color of the seed [16]. Color cameras are employed to capture the image of the seed and compare the color values with data stored in the lookup table to accept or reject. The disadvantage of the above stated systems is that these complex operating algorithms can provide only surface information and are incapable of analyzing the internal morphology of the seeds. Internal seed structural analysis can enable the user to determine the seed growth during the seed priming process, fungus growth in disease infected seeds, and internal morphological changes in seeds during the shooting stages, etc. [17,18,19,20]. Hence, an optical detection technique with multifunctional capability is required to analyze the internal structure of the seed and to differentiate seed specimens stained with different dye agents simultaneously.

Optical coherence tomography (OCT) is one of the NIR imaging modalities based on low coherence interferometry, which has a plethora of applications in biomedical imaging and diagnostics. It has the potential to perform real-time, non-destructive, and high resolution two dimensional (2D) cross-sectional imaging and three dimensional imaging (3D) of samples aimed at improved diagnosis [21]. Owing to its ability to perform high resolution real-time imaging, numerous biomedical research and medical imaging fields have been explored, such as ophthalmology [22], dentistry [23], otolaryngology [24], oncology [25], gastroenterology [26],dermatology [27,28], and in research studies for industrial inspection [29,30,31,32]. It can image with an axial resolution of 1–15 μm [33]. In the past few decades, OCT has emerged as a powerful tool in plant material study and has contributed much towards several agricultural researches [34,35,36]. The first application of OCT for agricultural research was demonstrated through the OCT imaging of internal seed structure of plant tissue and in vivo monitoring of plant tissue regeneration at different water supply conditions in 2004. Several studies showing the application of OCT to analyze the hull thickness and to monitor roots and the defects of onion have been reported [34]. Additionally, our group has performed numerous OCT applications in agricultural studies for seed phenotyping, viral infection detection in seeds, early disease detection in seeds and leaves of plants, and germination rate monitoring, as reported recently [17,18,19,20,37,38]. Our group’s research has focused on proposals and implementations of new novel applications of OCT for farmers and other agronomy researchers for monitoring of disease growth in plants and its evaluation, implementation of OCT systems to determine the plant health, and growth monitoring. Also, our research explores the use of compact and portable OCT systems for field studies. Earlier studies on seed priming, fungal growth and its effect on seed growth were focused just before or after planation of seeds. The present study focuses on the applicability to use OCT systems before the seed plantation; i.e., seed assessment and evaluation before the sowing process.

Hence, the aim of this study is to demonstrate the application of the SS-OCT system as a multipurpose imaging technique for the sorting of diversely stained seeds and as a non-destructive internal structural analysis of *C. annuum* seeds, which cannot be attained using conventional sorting techniques. Changes in the intensity of back-scattered light of the acquired OCT images from the control samples and the diversely stained seed specimens (resulting due to dye pigment material and internal morphological variations) were further analyzed by plotting back-scattered light intensities in the OCT images along depth direction to gain a precise confirmation of the concurred results from the visual inspection of OCT images. Thus, the proposed applicability of SS-OCT as a non-destructive tool in the agricultural industries to differentiate diversely stained seeds and to observe the internal morphology of seeds concurrently will be helpful in agricultural industries and for agronomical research.

## 2. Materials and Methods

### 2.1. Swept Source System Configuration

**Figure 1:**
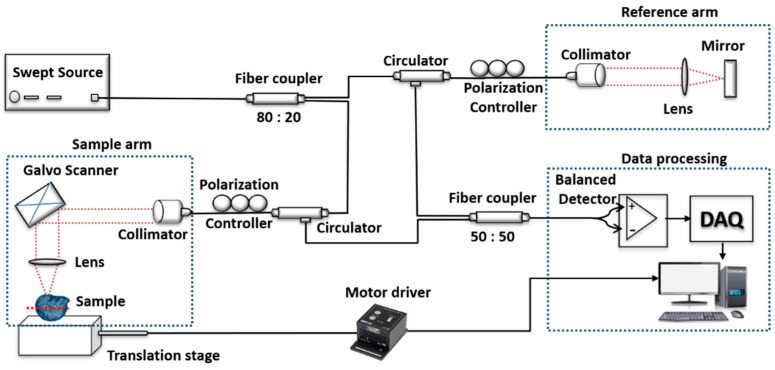
Schematic diagram of the swept-source optical coherence tomography (OCT) system configuration. DAQ: Data acquisition.

Figure 1 shows the schematic of the SS-OCT system utilized in this study. The entire experiment setup consists of a commercial SS-OCT system (OCS1310V1, Thorlabs. Inc, Newton, NJ, USA) and a motorized stage (PT1/M-Z8, Thorlabs. Inc, Newton, NJ, USA) placed perpendicularly underneath the sample arm for fast acquisition and serial scanning of seed specimens, which maintains a constant seed orientation. The commercial SS-OCT system was powered by a microelectromechanical system based vertical-cavity surface-emitting laser (MEMS-VCSEL) swept source laser centered at 1300 nm ± 15 nm with a spectral bandwidth of 97 nm (−10 dB cut off point) was used to obtain a depth resolution of 16 μm in air. The axial scan rate is 100 KHz, coherence length >50 mm, with an average output power of 20 mW. The laser source is connected to one of the input ports of the 80:20 fiber coupler. Twenty percent of the input source power is fed to the reference arm through a circulator and remaining eighty percent of power is fed to the sample arm with the help of another circulator. A coupler with the unbalanced split ratio is used to improve the power efficiency and SNR of the system relative to the standard design [39]. The reference arm is made of a collimator, lens, and a mirror as shown in Figure 1. Dual-axis galvanometer and an objective lens (LSM03, Thorlabs. Inc., Newton, NJ, USA) of numerical aperture (N.A) 0.055 with effective focal length (EFL) of 36 mm and depth of view (DOV) of 0.58 mm constituted the sample arm setup. The final optical power measured at sample arm end was 14.2 mW. These dual-axis galvanometer mirrors enable cross-sectional as well as volumetric imaging of the sample. The backscattered light from both sample and reference arms interfere at the 50:50 fiber coupler. The interference signal at the output of this coupler is connected to the positive and negative port of the balanced photodetector. The output signal from the photodetector is fed to a high-speed 12-bit digitizer with a sampling frequency of 4 GHz. A graphical user interface algorithm containing the background subtraction, k-domain linearization based on k-clock, fast Fourier transformation, and intensity log scaling processes the raw data to obtain the OCT images. The acquired B-scan (2D image) data after processing consists of 2000 A-scans (depth scans) with each A-scan consisting of 1407 pixels. The three-dimensional image (C-scan) was formed by 500 successive B-scans. The axial and transverse resolutions measured in the air are 16 and 25 μm respectively. The system offers a maximum imaging width of 10 mm and intensity fall off up to 12 mm, and the sensitivity of the system was 111 dB at zero optical-path length difference (OPD), and 73 dB at 12 mm.

### 2.2. Seed Sample Preparation

All the *C. annuum* seed specimens used for this experiment were collected from the Institute for Microorganism, Kyungpook National University, Daegu, South Korea. The total number of seeds used for this experiment was forty, which was divided into four groups with ten samples per each group. Seed category 1 represents the unstained *C. annuum* seeds from freshly extracted *C. annum* fruit. It served as the control sample. The other three seed categories represent *C. annuum* seeds, which are uniformly dye stained belonging to three different cultivar varieties, *C. annuum* cv. PR Ppareum, *C. annuum* cv. PR Yeol, and *C. annuum* cv. Asia Jeombo, respectively. *C. annuum* cv. PR Ppareum seeds belonging to the seed category 2 were stained with blue dye. *C. annuum* cv. PR Yeol seeds representing the seed category 3, which were stained with green dye, and the final seed category 4 of *C. annuum* cv. Asia Jeombo were stained with red dye. The diameter of all the seeds selected was in the range of 4 to 5 mm. The freshly extracted seeds were dried for 2 days at room temperature (24 °C) prior to imaging, and all the seeds selected for this study were uniformly matured.

### 2.3. Experimental Algorithm

**Figure 2:**
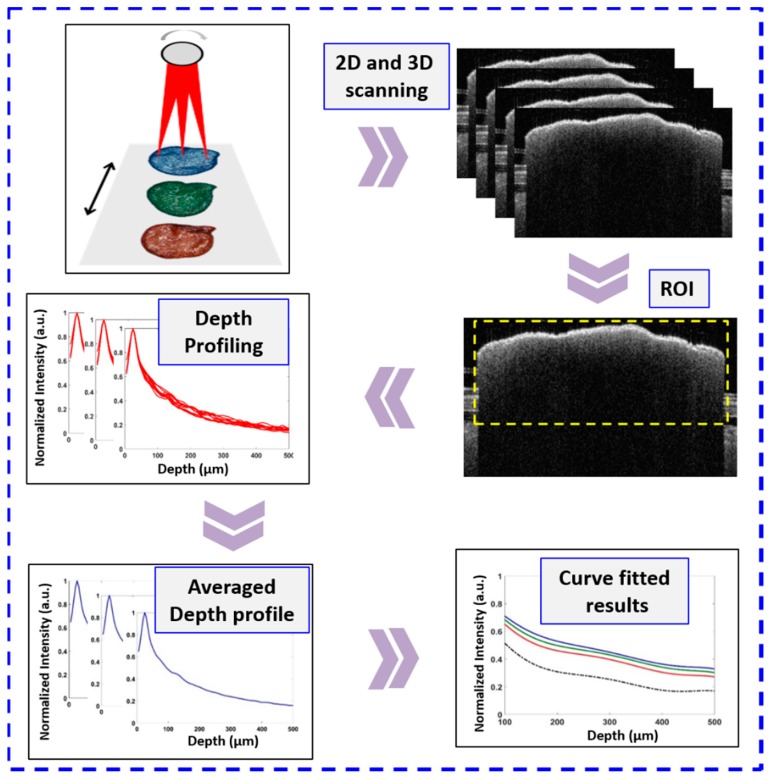
A flowchart explaining the experimental algorithm used to obtain the depth intensity profile in different seed samples.

Figure 2 depicts the flowchart explaining the experimental algorithm. Prior to imaging, all the ten samples from each seed category were arranged in a similar fashion on the translation stage to provide the same seed orientation during the entire imaging process. Figure 1 shows the exact location and orientation of the sample on the translation stage during imaging. The seed’s radical emergence point was placed facing forward towards the OCT scanning sample arm during imaging. The red dashed line on the seed sample in Figure 1 shows the exact scan position. The focusing point of the sample arm was maintained just below the second most layer from the top surface of the seed, this was achieved by moving the sample arm up or down accordingly while visually confirming from the real-time 2D OCT image. This was followed for all the samples so that the seeds were imaged within the focusing depth range of the sample arm. All the samples were imaged in a sequential manner with the help of a program controlled motorized stage. This was done to achieve a faster image acquisition, with a specific end goal to lessen the impact of power fluctuation in the laser source over the prolonged imaging period as in conventional imaging process for experiments involving large sample numbers. A scanning range of 5 mm × 5 mm and the refractive index of 1.42 of seed was used throughout the imaging process. This was repeated with samples from all the 4 categories. The total time taken for the volumetric scanning was 5 s.

The acquired OCT cross sectional images were further analyzed using depth scan profile plotting. The depth scan analysis was performed over a uniform region of interest (approximately 1000 A-scans) on all the forty acquired 2D-OCT images using a MATLAB (Mathworks, Middlesex County, NJ, USA) coded program. The region of interest is highlighted in Figure 2 with a yellow dashed rectangle. The acquired OCT images are not flattened, the maximum back-scattered light intensity points are indexed at different positions because of the physical structure of the seed. Thus, the code detects the maximum back-scattered light intensity peak from 1000 A-scans selected from the whole B-scan image and are rearranged and matched linearly to flatten the image.

The flattened image consisting of the 100 A-scans (consecutive adjacent 100 A-scan signals from the first A-scan signal within the selected window) are then averaged to reduce the effect of noise in the depth profile plots. This averaged depth scan was normalized by dividing by the maximum intensity values, to represent the depth scan profile of each seed sample. Using a similar procedure, the depth scan analysis was performed on all the acquired 2D OCT images. To obtain an averaged depth profile plot for each seed, successive multiple A-scans in each of the 2D images were averaged. Later, an averaged depth scan from each seed category was obtained by averaging the depth scans of all ten seeds from each category. This averaged depth scan was used to represent each seed category and the variations in back-scattered intensities of each seed (seed 1–10) when averaged for each seed group category were taken to be the standard deviation. The averaged depth scans were further curve fitted. Curve fitting is commonly done to theoretically describe the experimental data with a model for smoothening the data. Here, curve fitted plot for each variety was obtained by fitting the averaged depth scans to a polynomial of 6th order. We chose the 6th order degree of polynomial to get maximum correlation coefficient (R) of the plotted graphs. The MATLAB code for curve fitting comprises of “polyfit” and “polyval” functions. To measure the correlation coefficient (R), the “corrcoef” function was utilized. The representative average depth scans and curve fitted plots were further compared with one seed category to other seed categories to observe the intensity difference between the different seed categories. This back-scattered light intensity difference laid the foundation for differentiation of different dye stained seeds. This intensity differences between the four seed categories observed in the curve fitted plots are then tabulated for comparative analysis.

## 3. Results and Discussions

**Figure 3:**
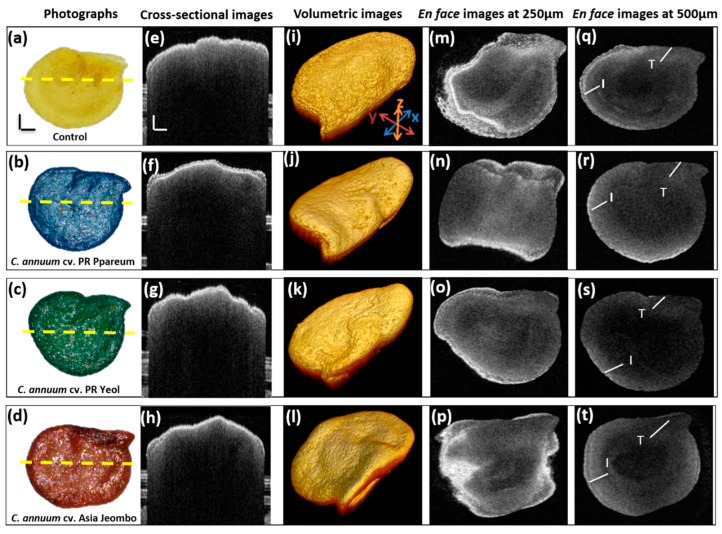
Photographs and OCT images of the samples used in this study. (**a**–**d**) Photographs of *Capsicum annuum* seeds belonging to cultivar varieties *C. annuum* (control), *C. annuum* cv. PR Ppareum, *C. annuum* cv. PR Yeol and *C. annuum* cv. Asia Jeombo varieties, respectively. (**e**–**h**) Corresponding 2D cross sectional images. (**i**–**l**), (**m**–**p**) and (**q**–**t**) show their corresponding 3D cross sectional images, *en face* images at depth of 250 and 500 μm, respectively. I: Inner seed coat; T: Testa. The horizontal and vertical scale bar of the figure: 500 μm.

The seeds were imaged two-dimensionally and three-dimensionally using a 1300 nm SS-OCT system and the acquired OCT images were used for analysis purposes. Figure 3 shows the external appearance and OCT images of the samples used. Figure 3a represents the photograph of the unstained *C. annum* control seed sample. Figure 3b–d show the photographs of dye stained seeds belonging to *C. annuum* cv. PR Ppareum, *C. annuum* cv. PR Yeol, and *C. annuum* cv. Asia Jeombo variety, respectively. All the photographs shown in the figure were taken with USB microscope with 30× magnification. Figure 3e–h represent the 2D OCT cross-sectional images of unstained *C. annuum* seeds, *C. annuum* cv. PR Ppareum stained with blue dye, *C. annuum* cv. PR Yeol stained with the green dye, and *C. annuum* cv. Asia Jeombo stained with red dye, in order. The acquired OCT cross-sectional images were first analyzed to see the difference among the four categories. Comparing the Figure 3e–h visually, no significant difference in the depth information among the seed categories was observed. Only the Testa and Inner seed coat were visible in all the cross-sectional images. The two distinct visible layers are marked in the same figure as T: Testa and I: Inner seed coat. The images do not exhibit much information on the internal seed regions, such as embryo, endosperm or storage cotyledons.

Figure 3i–l shows the 3D OCT images of the representative seeds. All 3D images were processed and their corresponding *en face* images taken at a uniform depth of 250 and 500 μm from the seed surface as shown in Figure 3m–t, respectively. The *en face* images provide the frontal view of the inner seed structure. The *en face* images at a depth of 250 and 500 μm were taken to study in detail the internal layer information of the seed. Comparing the *en face* images of all seed categories, there were few observable changes in the internal structure among the seed categories. At 250 μm depth, all the seed categories gave almost similar information. The seed category 2, stained with the blue dye gave no significant information about the internal structures. Only the outer seed coat and the Testa were clearly distinguishable. The seed category 4, the seeds stained with the red dye and the control samples gave similar layer information. Other than the outer seed coat and Testa, one more inner layer was visible. Seed category 3, stained with green dye, gave better inner structure information than seed category 1, but less information than seed category 4. This was clearer in the 500 μm *en face* images. These observations were further analyzed using depth scan profiles.

**Figure 4:**
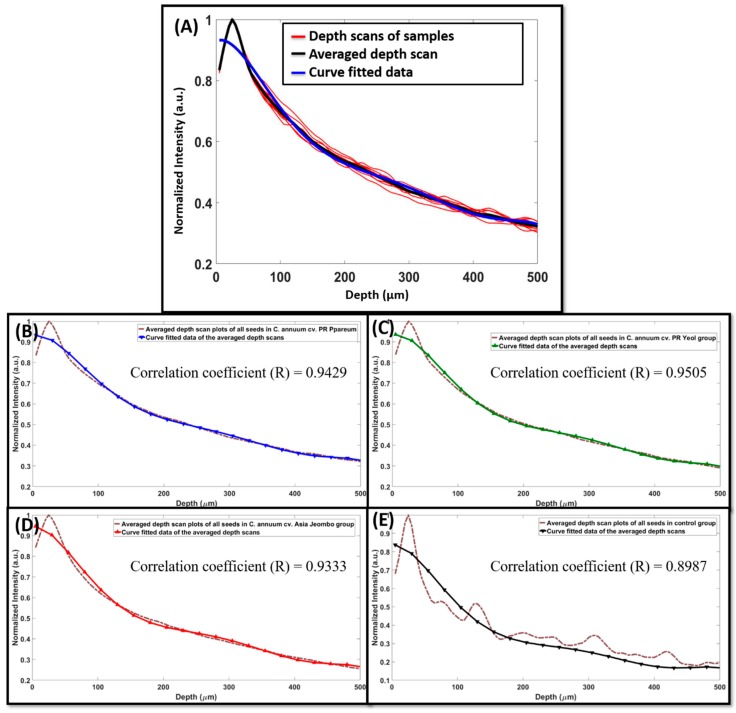
Curve fitting and correlation coefficient measurement. (**A**) is normalized depth scan profile and curve fitted plot of *Capsicum annuum* cv. PR Ppareum. It shows the normalized depth scan profiles (red) of all 10 samples, averaged depth scan (black), and curve fitted data (blue) of control category seeds. (**B**–**E**) are the averaged and curve fitted plots of all four groups, the correlation coefficient values are shown in the respective plots.

The depth scan profiles for the acquired OCT images were taken as mentioned in the previous section. Figure 4A shows the overlapped plot of the individual depth scans of the ten samples, the representative depth scan obtained by averaging the ten individual depth scans, and the curve fitted data of the averaged depth scans of one selected seed category, represented in red, blue color, and black color, respectively. Figure 4B–E are the averaged plots and the respective curve fitted plots of all four groups. The measured correlation coefficient (R) for the groups *C. annuum* cv. PR Ppareum, *C. annuum* cv. PR Yeol, *C. annuum* cv. Asia Jeombo, and the control were 0.9429, 0.9505, 0.9333, and 0.8987 respectively. It was observed that the individual depth scan profile gave very slight variation from the averaged depth scans, validating the use of averaged depth scan for representing an individual seed category. The averaged depth scans were further curve fitted. Curve fitting is done to theoretically describe the experimental data with a model for data smoothening. Here, a curve fitted plot for each variety was obtained by fitting the averaged depth scans to a polynomial of the 6th order. This was repeated for all the four seed categories. The intricate pattern in the averaged depth scan plot of all the seeds in the control group is unique and shows more intensity fluctuations when compared to other seed categories. This could be due to the high scattering of light from the superficial layer of seed samples by dye pigments in the dye treated seed category groups, which is absent in control samples (without dye treated). Hence, more fluctuations of intensity correlate to internal structures of the seeds in control samples.

**Figure 5:**
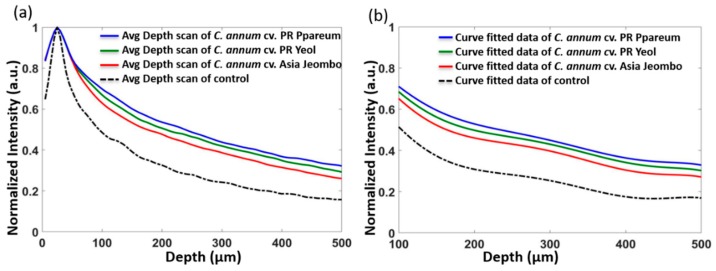
A Comparison of averaged depth scan profiles and the curve fitted data of seed categories. (**a**) Averaged depth scan profiles of *C. annuum* cv. PR Ppareum, *C. annuum* cv. PR Yeol, *C. annuum* cv. Asia Jeombo and control (**b**) Curve fitted data plotted from the depth of 100 to 500 μm of all seed categories.

**Figure 6:**
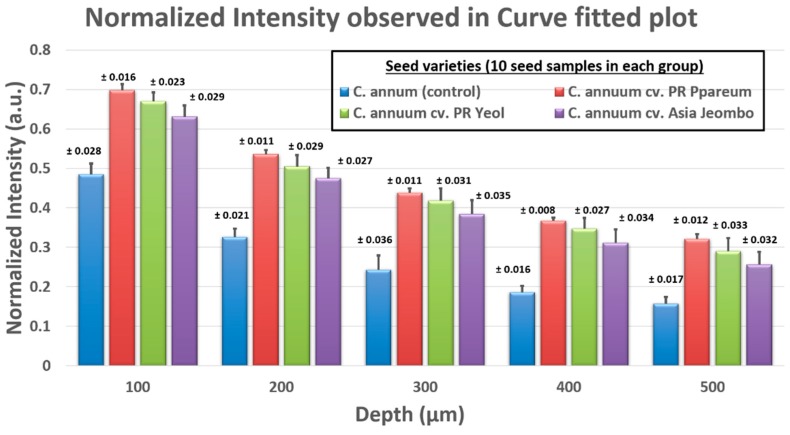
Graphical representation of the normalized intensities observed in curve fitted plot at different depth for all seed category groups. The detailed representation of values is shown in Table 1.

Figure 5a depicts the averaged depth scan profiles of *C. annuum* cv. PR Ppareum, *C. annuum* cv. PR Yeol, *C. annuum* cv. Asia Jeombo, and unstained control *C. annuum* seeds shown in a single graph. Averaged depth scan profiles of *C. annuum* cv. PR Ppareum, *C. annuum* cv. PR Yeo and *C. annuum* cv. Asia Jeombo seeds were represented using blue, green, and red colored solid lines, respectively. The unstained *C. annuum* control samples were represented by a black colored dashed line. The averaged depth scans showed a significant back-scattered light intensity difference between the control sample and the dye stained samples. This intensity difference was observed over the complete depth. Among the dye stained samples *C. annuum* cv. PR Ppareum stained with blue dye gave maximum back-scattered light intensity images, followed by *C. annuum* cv. PR Yeol variety stained with the green dye and *C. annuum* cv. Asia Jeombo stained with red dye. These averaged depth scans were further curve fitted to verify the back-scattered light intensity difference. The curve fitted data was obtained for a depth range of 100 to 500 μm. Figure 5b compares the intensity difference in curve-fitted data of dye stained *C. annuum* cv. PR Ppareum, *C. annuum* cv. PR Yeol, *C. annuum* cv. Asia Jeombo, and unstained control *C. annuum* seeds represented in blue, green, red, and black color, respectively. From the curve fitted data, it is more evident that among the dye stained categories, the maximum back-scattered light intensity was observed in seed category of *C. annuum* cv. PR Ppareum stained with the blue dye. *C. annuum* cv. PR Yeol variety stained with the green dye gave slightly lower back-scattered light intensity images compared to *C. annuum* cv. PR Ppareum stained with the blue dye and slightly high-intensity images compared to *C. annuum* cv. Asia Jeombo stained with red dye. Minimum back-scattered light intensity images among the dye stained category were given by *C. annuum* cv. Asia Jeombo stained with red. The unstained control samples gave very low-intensity images compared to all other the dye stained samples. This is in line with observations from averaged depth scan profiles. The back-scattered light intensity variation between all the four categories, obtained at varying depths of 100, 200, 300, 400, and 500 μm is collectively shown in Table 1. The Table 1 values are the curve fitted normalized back-scattered intensity values that were observed at different depths. These averaged values in Table 1 were intensities that were measured in curve fitted plot for each seed category groups (10 seeds in each group). The detailed information of the back-scattered intensities of all seeds at five different depths (100 µm, 200 µm, 300 µm, 400 µm, and 500 µm) in all four seed category groups is given in the Supplementary Materials Tables S1–S4. For better visualization and easier understanding, a graphical representation (Figure 6) of the intensity variations in curve fitted plot and the standard deviation values shown in Table 1.

We can observe a very significant difference in the intensities of the unstained seed category represented by the black dashed line, from all the other dye stained categories. The maximum back-scattered light intensity image from *C. annuum* cv. PR Ppareum stained with blue dye can be due to the maximum backscattering from the superficial layers of seed samples. This high backscattering from the sample can be the most possible reason for reduced layer information in *en face* images. Similarly, the least back-scattered light intensity images from the control samples can be due to the comparatively less backscattering from the superficial layers of control samples. This could be the reason for more layer information from the *en face* images of control samples. This high back-scattered light intensity in images acquired from dye stained samples may be because of the difference in refractive index of the dye materials used.

In the current proposed method, the numerical analysis was done as a post processing. The main aim of this study was to determine if OCT can be used as an alternative tool for the machine-vison system, which is conventionally employed for the seed sorting mechanism. Furthermore, the advantages of OCT as a real-time, non-destructive cross-sectional imaging system may help in viewing the cross-sectional layer information [18,19,20,36,37]. The intensity plots for analyzing the superficial layer thickness and optical properties of the seed layers revealed few variations and changes when one seed category was compared to the other seed categories. We speculate the reason was that all seeds were almost similar in internal structure and moreover, the coating applied to the seeds was very thin and may have been thinner than the OCT system resolution to measure the coating/shell thickness. We know that the scattering and absorption of light greatly varies depending on the material properties and composition, and also on the color of the material. The colored pigments (in this case dye materials) only absorb a part of the wavelengths of the light that are exposed onto it. The material pigment composition in dyes varies depending on the type of dye material used, which depends on the preferences of cultivars. The optical scattering and absorption properties of light exposed on to the dyes (with different pigment composition in dyes that have the same color) may meagerly vary according to the pigment composition in the dye material. However, in general the overall scattering and absorption properties of light exposed on the dye varies greatly depending on the dye color. Furthermore, a specific wavelength range of light (for example, a near-infrared light of 1300 nm center wavelength source with a 100 nm bandwidth) will have a higher and distinct back-scattering of light when exposed on different colored dyes. This property can also be observed when a visible light source is used, but the amount of back-scattering of the total light intensities will be due to different sets of wavelength ranges in the visible spectrum. This makes it slightly difficult to determine which wavelength ranges contribute to the maximum back-scattering intensity. Furthermore, the direction of light travel differs on the refractive index of the particle and the surrounding material. In the case of dyes coated on seeds, the scattering of light differs greatly on the refractive index of the dye material and the seed. Also, the composition of the dye material, which gives its color, also has an effect on the scattering of light exposed on to it. This can be seen in our obtained results, where the dye colors of blue, green, and red are coated on the seeds. Furthermore, near-infrared laser source scatters accordingly to these dye colors and its material composition. In the aforementioned results we can see the blue dye coated *C. annuum* cv. PR Ppareum seeds shoed maximum back-scattered light intensity in the curve fitted normalized intensity plots, the second highest back-scattered light intensity in the curve fitted normalized intensity plots was found to be from the green dye coated *C. annuum* cv. PR Yeol seeds, and the red dye coated *C. annuum* cv. Asia Jeombo seeds had comparatively lesser back-scattered light intensity in the curve fitted normalized intensity plots. Finally, the least back-scattered light intensity in the curve fitted normalized intensity plots was found to be from the untreated freshly extracted from the *C. annum* fruit. These variations in intensities of different seed groups were due to the changes in absorption and scattering properties of the dye materials.

**Figure 7:**
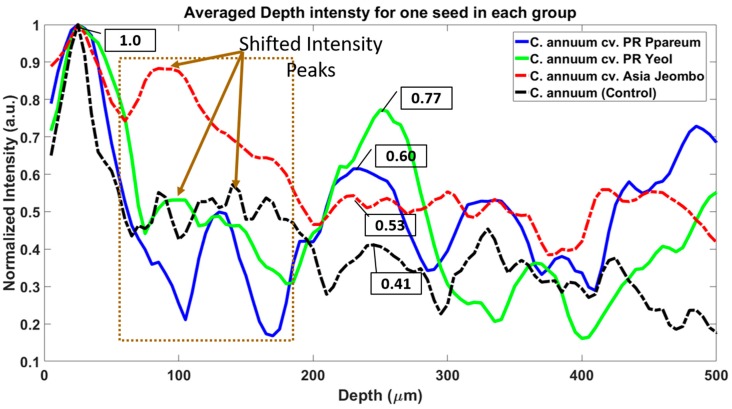
Averaged and normalized depth intensity plots of one seed in all four seed groups. The averaging window was set to 10 for all plots.

For measuring the difference in attenuation of samples using 1300 nm SS-OCT system, we took one 2D OCT image of one seed per group. We plotted the graph of the averaged and normalized depth intensity profile for these seeds and compared the reduced intensity values for each unique peak in the plotted profile. The averaging window was set to 10 for all 2D OCT images. This is shown in Figure 7. The variations in intensities of the samples are because of two reasons, one is the internal structural changes in the seeds, which can be seen in shifted and broadened intensity of the plotted graph (shown in dashed brown box and with brown arrows). Second is the back-scattered intensity variations, which arise due to the difference in dye material. The difference in the back-scattered intensity in peaks, which are almost in the same position in the depth direction (x-axis), is due to the difference in color and pigments in the dye material.

## 4. Conclusions

In the proposed study, the swept source OCT system was used to image different dye stained *Capsicum* annum seeds and the OCT images of different dye stained *C. annum* seeds belonging to different cultivar varieties, such as *C. annuum* cv. PR Ppareum, *C. annuum* cv. PR Yeol and *C. annuum* cv. Asia Jeombo were analyzed and compared with unstained *C. annum* seeds from freshly extracted fruit. The acquired two-dimensional and three-dimensional images were unable to differentiate between the different dye stained seed samples, hence, a depth scan profile analysis was carried out for all the acquired OCT images. By analyzing the normalized depth intensity scan profiles, a significant difference in the intensities of OCT images between unstained seed samples and different dye stained seed samples was observed. The variedly dye stained seed specimens exhibited different back-scattered light intensity images when imaged under the same circumstances. These intensity differences in images could have been as a result of the varying refractive index of the dye materials used. Our experimental result confirms the potential of depth scan back-scattered light intensity profiles of OCT images for differentiating different dye stained *C. annum* seed samples. This back-scattered light intensity detection capability of depth profiles can be used in agricultural industries to differentiate the seeds that are differently stained. When compared to conventional seed sorting techniques, OCT offers multipurpose advantages by performing the sorting of seeds in respective to the dye treatment and provides internal structural images non-destructively. The main aim of this study was to evaluate if the OCT system can be used for distinguishing the seeds by seed category of cultivars that were stained using different dyes. To our knowledge, this study is the first of its kind, which demonstrates OCT system capability for distinguishing seeds based on staining. Furthermore, in the future OCT incorporated with a vision system can be used in the large-scale agriculture industries to improve the seed monitoring and seed sorting techniques for agronomical research.

## Figures and Tables

**Table 1:** Normalized intensity observed for control, *C. annuum* cv. PR Ppareum, *C. annuum* cv. PR Yeol and *C. annuum* cv. Asia Jeombo seed varieties at different depths in curve fitted plot derived from OCT images.

Normalized Intensity Observed at Different Depths (a.u.)										
Seed Varieties (10 Seed Samples in Each Group)	Depth (µm)									
	100 (µm)		200 (µm)		300 (µm)		400 (µm)		500 (µm)	
	Average	Standard Deviation	Average	Standard Deviation	Average	Standard Deviation	Average	Standard Deviation	Average	Standard Deviation
**C. annum (control)**	0.485	0.028	0.326	0.021	0.243	0.036	0.186	0.016	0.157	0.017
**C. annuum cv. PR Ppareum**	0.698	0.016	0.536	0.011	0.438	0.011	0.367	0.008	0.321	0.012
**C. annuum cv. PR Yeol**	0.670	0.023	0.505	0.029	0.418	0.031	0.347	0.027	0.290	0.033
**C. annuum cv. Asia Jeombo**	0.631	0.029	0.475	0.027	0.384	0.035	0.311	0.034	0.256	0.032

## Supplementary Materials

The following are available online at http://www.mdpi.com/1424-8220/18/8/2500/s1, Table S1: Normalized Intensity observed at different depths for all seeds in *C. annum* group (control), Table S2: Normalized Intensity observed at different depths for all seeds in *C. annuum* cv. PR Ppareum group, Table S3: Normalized Intensity observed at different depths for all seeds in *C. annuum* cv. PR Yeol group, Table S4: Normalized Intensity observed at different depths for all seeds in *C. annuum* cv. Asia Jeombo group.
