# Supplementary material for: Non-Destructive Classification of Diversely Stained Capsicum annuum Seed Specimens of Different Cultivars Using Near-Infrared Imaging Based Optical Intensity Detection
Source: Sensors (Basel). 2018 Aug 1;18(8):2500. doi: 10.3390/s18082500 (PMC6111981; doi:10.3390/s18082500)
Supplement: Supplementary file 1 [file sensors-18-02500-s001.pdf]

sensors

Supplementary Materials

Non-Destructive Classification of Diversely Stained *Capsicum annuum* Seed Specimens of Different Cultivars Using Near-Infrared Imaging Based Optical Intensity Detection

Jyothsna Konkada Manattayil [1,†], Naresh Kumar Ravichandran [2,†], Ruchire Eranga Wijesinghe [3], Muhammad Faizan Shirazi [4], Seung-Yeol Lee [5], Pilun Kim [6], Hee-Young Jung [5,*], Mansik Jeon [2,*] and Jeehyun Kim [2]

1 Department of Electronics and Communication, Faculty of Engineering, Christ (Deemed to be University), Bangalore 560029, India; jyothsna.km@mtech.christuniversity.in
2 Kyungpook National University, College of IT Engineering, School of Electronics Engineering, 80, Daehak-ro, Buk-gu, Daegu 41566, Korea; nareshr.9169@gmail.com (N.K.R.); jeehk@knu.ac.kr (J.K.)
3 Department of Biomedical Engineering, College of Engineering, Kyungil University, 50, Gamasil-gil, Hayang-eup, Gyeongsan-si, Gyeongsangbuk-do 38428, Korea; eranga@kiu.kr
4 Center of Medical Physics and Biomedical Engineering, Medical University of Vienna, Waehringer Guertel 18-20, 1090 Vienna, Austria; muhammad.shirazi@meduniwien.ac.at (M.F.S.)
5 School of Applied Biosciences, Kyungpook National University, 80, Daehak-ro, Buk-gu, Daegu 41566, Korea; leesy1985@gmail.com
6 Institute of Biomedical Engineering, Kyungpook National University, 680, Gukchaebosang-ro, Jung-gu, Daegu 41944, Korea; pukim@knu.ac.kr
* Correspondence: heeyoung@knu.ac.kr (H.-Y.J.); msjeon@knu.ac.kr (M.J.); Tel.: +82-53-950-7846 (M.J.)
† These authors contributed equally to this work.

Table S1. Normalized Intensity observed at different depths for all seeds in *C. annum* group (control).

Normalized Intensity Observed at different depths (a.u.)						
		Depth (µm)				
		100	200	300	400	500
C. annum (control)	seed1	0.5038	0.3344	0.238	0.1856	0.1439
	seed2	0.4898	0.3251	0.2295	0.2013	0.1614
	seed3	0.4983	0.3127	0.2199	0.1731	0.1421
	seed4	0.5258	0.3445	0.2477	0.1911	0.1791
	seed5	0.4756	0.2972	0.2206	0.1741	0.1515
	seed6	0.4744	0.3037	0.2207	0.1685	0.1513
	seed7	0.4431	0.3588	0.3429	0.2242	0.1938
	seed8	0.4393	0.3141	0.2259	0.1792	0.1526
	seed9	0.4838	0.3098	0.2266	0.1768	0.1353
	seed10	0.5215	0.3565	0.2607	0.1844	0.1617
	Average	0.48554	0.32568	0.24325	0.18583	0.15727
	Standard deviation	0.02755649	0.02079989	0.03551907	0.01570643	0.01682255

Table S2. Normalized Intensity observed at different depths for all seeds in *C. annuum* cv. PR Ppareum group.

Normalized Intensity Observed at different depths (a.u.)						
C. annuum cv. PR Ppareum		Depth (µm)				
		100	200	300	400	500
	seed1	0.69	0.5267	0.4366	0.36	0.315
	seed2	0.7321	0.5568	0.4484	0.3745	0.3314
	seed3	0.6953	0.5404	0.4577	0.3763	0.324
	seed4	0.7131	0.5501	0.4478	0.3798	0.3228
	seed5	0.7088	0.5429	0.4394	0.3711	0.3377
	seed6	0.6948	0.5309	0.4318	0.3554	0.3074
	seed7	0.6707	0.5167	0.4281	0.3604	0.3386
	seed8	0.6832	0.5325	0.4377	0.3696	0.3246
	seed9	0.7051	0.5363	0.4376	0.3673	0.3124
	seed10	0.6886	0.5255	0.4155	0.3566	0.3026
	Average	0.69817	0.53588	0.43806	0.3671	0.32165
	Standard deviation	0.01640671	0.01142741	0.01113159	0.00815733	0.01162439

Table S3. Normalized Intensity observed at different depths for all seeds in *C. annuum* cv. PR Yeol group.

Normalized Intensity Observed at different depths (a.u.)						
C. annuum cv. PR Yeol		Depth (µm)				
		100	200	300	400	500
	seed1	0.636	0.4894	0.4033	0.327	0.2876
	seed2	0.6433	0.4809	0.4069	0.3356	0.2667
	seed3	0.6817	0.472	0.3964	0.3288	0.2735
	seed4	0.6527	0.5007	0.401	0.3362	0.2621
	seed5	0.6448	0.4841	0.4004	0.3283	0.2697
	seed6	0.6845	0.5034	0.3877	0.3195	0.2611
	seed7	0.6697	0.4797	0.3898	0.3334	0.2721
	seed8	0.6944	0.5467	0.4662	0.384	0.333
	seed9	0.6953	0.5544	0.4725	0.4006	0.363
	seed10	0.7001	0.5412	0.4563	0.3784	0.3137
	Average	0.67025	0.50525	0.41805	0.34718	0.29025
	Standard deviation	0.02300279	0.02915508	0.03142977	0.02737944	0.03302436

Table S4. Normalized Intensity observed at different depths for all seeds in *C. annuum* cv. Asia Jeombo group.

Normalized Intensity Observed at different depths (a.u.)						
		Depth (μm)				
		100	200	300	400	500
C. annuum cv. Asia Jeombo	seed1	0.6113	0.4433	0.3664	0.2965	0.23
	seed2	0.5979	0.454	0.341	0.2622	0.2153
	seed3	0.6898	0.5378	0.4733	0.3983	0.3394
	seed4	0.615	0.469	0.3575	0.2924	0.2425
	seed5	0.6329	0.4774	0.406	0.3325	0.2687
	seed6	0.6457	0.466	0.373	0.2943	0.2366
	seed7	0.631	0.4861	0.3873	0.3052	0.2647
	seed8	0.6299	0.4467	0.3549	0.3036	0.2464
	seed9	0.587	0.463	0.3975	0.3209	0.2646
	seed10	0.6653	0.5034	0.3832	0.3074	0.2532
	Average	0.63058	0.47467	0.38401	0.31133	0.25614
	Standard deviation	0.02920407	0.02717731	0.03532858	0.03390923	0.03202718
